# Longevity is determined by ETS transcription factors in multiple tissues and diverse species

**DOI:** 10.1371/journal.pgen.1008212

**Published:** 2019-07-29

**Authors:** Adam J. Dobson, Richard Boulton-McDonald, Lara Houchou, Tatiana Svermova, Ziyu Ren, Jeremie Subrini, Mireya Vazquez-Prada, Mimoza Hoti, Maria Rodriguez-Lopez, Rita Ibrahim, Afroditi Gregoriou, Alexis Gkantiragas, Jürg Bähler, Marina Ezcurra, Nazif Alic

**Affiliations:** 1 Institute of Healthy Ageing, Department of Genetics, Evolution and Environment, University College London, London, United Kingdom; 2 School of Biosciences, University of Kent, Canterbury, United Kingdom; Princeton, UNITED STATES

## Abstract

Ageing populations pose one of the main public health crises of our time. Reprogramming gene expression by altering the activities of sequence-specific transcription factors (TFs) can ameliorate deleterious effects of age. Here we explore how a circuit of TFs coordinates pro-longevity transcriptional outcomes, which reveals a multi-tissue and multi-species role for an entire protein family: the E-twenty-six (ETS) TFs. In *Drosophila*, reduced insulin/IGF signalling (IIS) extends lifespan by coordinating activation of *Aop*, an ETS transcriptional repressor, and *Foxo*, a Forkhead transcriptional activator. *Aop* and *Foxo* bind the same genomic loci, and we show that, individually, they effect similar transcriptional programmes *in vivo*. In combination, *Aop* can both moderate or synergise with *Foxo*, dependent on promoter context. Moreover, *Foxo* and *Aop* oppose the gene-regulatory activity of *Pnt*, an ETS transcriptional activator. Directly knocking down *Pnt* recapitulates aspects of the *Aop*/*Foxo* transcriptional programme and is sufficient to extend lifespan. The lifespan-limiting role of *Pnt* appears to be balanced by a requirement for metabolic regulation in young flies, in which the *Aop-Pnt-Foxo* circuit determines expression of metabolic genes, and *Pnt* regulates lipolysis and responses to nutrient stress. Molecular functions are often conserved amongst ETS TFs, prompting us to examine whether other *Drosophila* ETS-coding genes may also affect ageing. We show that five out of eight Drosophila ETS TFs play a role in fly ageing, acting from a range of organs and cells including the intestine, adipose and neurons. We expand the repertoire of lifespan-limiting ETS TFs in *C*. *elegans*, confirming their conserved function in ageing and revealing that the roles of ETS TFs in physiology and lifespan are conserved throughout the family, both within and between species.

## Author summary

Understanding the basic biology of ageing may help us to reduce the burden of ill-health that old age brings. Ageing is modulated by changes to gene expression, which are orchestrated by the coordinate activity of proteins called transcription factors (TFs). E-twenty six (ETS) TFs are a large family with cellular functions that are conserved across animal taxa. In this study, we examine a longevity-promoting transcriptional circuit composed of two ETS TFs, *Pnt* and *Aop*, and *Foxo*, a forkhead TF with evolutionarily-conserved pro-longevity functions. This leads us to demonstrate that the activity of the majority of ETS TFs in multiple tissues and even different animal taxa regulates lifespan, indicating that roles in ageing are a general feature of this family of transcriptional regulators.

## Introduction

Ageing is characterised by a steady systematic decline in biological function, and increased likelihood of disease[1]. Understanding the basic biology of ageing therefore promises to help improve the overall health of older people, who constitute an ever-increasing proportion of our populations. In experimental systems, healthy lifespan can be extended by altered transcriptional regulation, coordinated by sequence-specific TFs[2–6]. Thus, understanding TFs’ functions can reveal how to promote health in late life. Forkhead family TFs, especially Forkhead Box O (*Foxo*) orthologues, have been studied extensively in this context. This effort has been driven by the association of *Foxo3a* alleles with human longevity[7]; and the findings that the activation of Foxos is necessary and sufficient to explain the extension of lifespan observed following reduced insulin/IGF signalling (IIS) in model organisms[8–11]. Foxos interact with additional TFs in regulatory circuits, and it is in this context that their function must be understood. For example, in *Caenorhabditis elegans*, the pro-longevity activity of *Daf-16* is orchestrated with further TFs including *Hsf*, *Elt-2*, *Skn-*1, *Pqm-1* and *Hlh-30/Tfeb* [3,12–15]. Examining regions bound by Foxos across animals has highlighted the conserved presence of sites to bind ETS family TFs[16]. In *Drosophila*, two members of this family, namely *Aop* (a.k.a. *Yan*) and *Pnt*, have been linked to ageing via genetic interactions with *Foxo* and IIS[4], and similar interactions are evident in *C*. *elegans* [17]. These findings raise questions of the overall roles of ETS factors in ageing, and their relationship to the activities of Foxos.

The ETS TFs are conserved across animals, including 28 representatives in humans[18,19]. Their shared, defining feature is a core helix-turn-helix DNA-binding domain, which binds DNA on 5’-GGA(A/T)-3’ ETS-binding motifs (EBMs). They are differentiated by tissue-specific expression, and variation in peripheral amino acid residues which, along with variation in nucleotides flanking the core EBM, confers DNA-binding specificity[20]. ETS TFs generally function as transcriptional activators, but a few repress transcription[21,22]. *Aop* is one such repressor in *Drosophila*. *Aop* and its human orthologue *Tel* are thought to repress transcription by competing with activators for binding sites[21,23], recruiting co-repressors[22,24,25], and forming homo-oligomers that limit activator access to euchromatin[26–30]. Consequently, *Aop*'s role in physiology must be explored in the context of its interactions with additional TFs, especially activators. *Foxo* is one such activator[31]. Both *Foxo* and *Aop* are required for longevity by IIS inhibition[9], each is individually sufficient to extend lifespan[4], and both are recruited to the same genomic loci *in vivo*. Whilst activating either in the gut and fat body extends lifespan, the effect of activating both is not additive. Furthermore, if *Aop* is knocked down, activating *Foxo* not only ceases to extend lifespan, but even becomes deleterious for lifespan[4]. Overall, these findings suggest that gene expression downstream of IIS is orchestrated by the coordinated activity of *Aop* and *Foxo*, and that there is a redundancy in the function of the two TFs, even though *Foxo* is a transcriptional activator and *Aop* a transcriptional repressor. We started this study by characterising *Aop* and its relationship with relevant transcriptional activators, including *Foxo*. This led us to uncover that roles in ageing are widespread throughout the ETS TF family, extending across multiple fly tissues and diverse animal taxa.

## Results

### AOP orchestrates an equivalent transcriptional programme to FOXO *in vivo*

**Fig 1:**
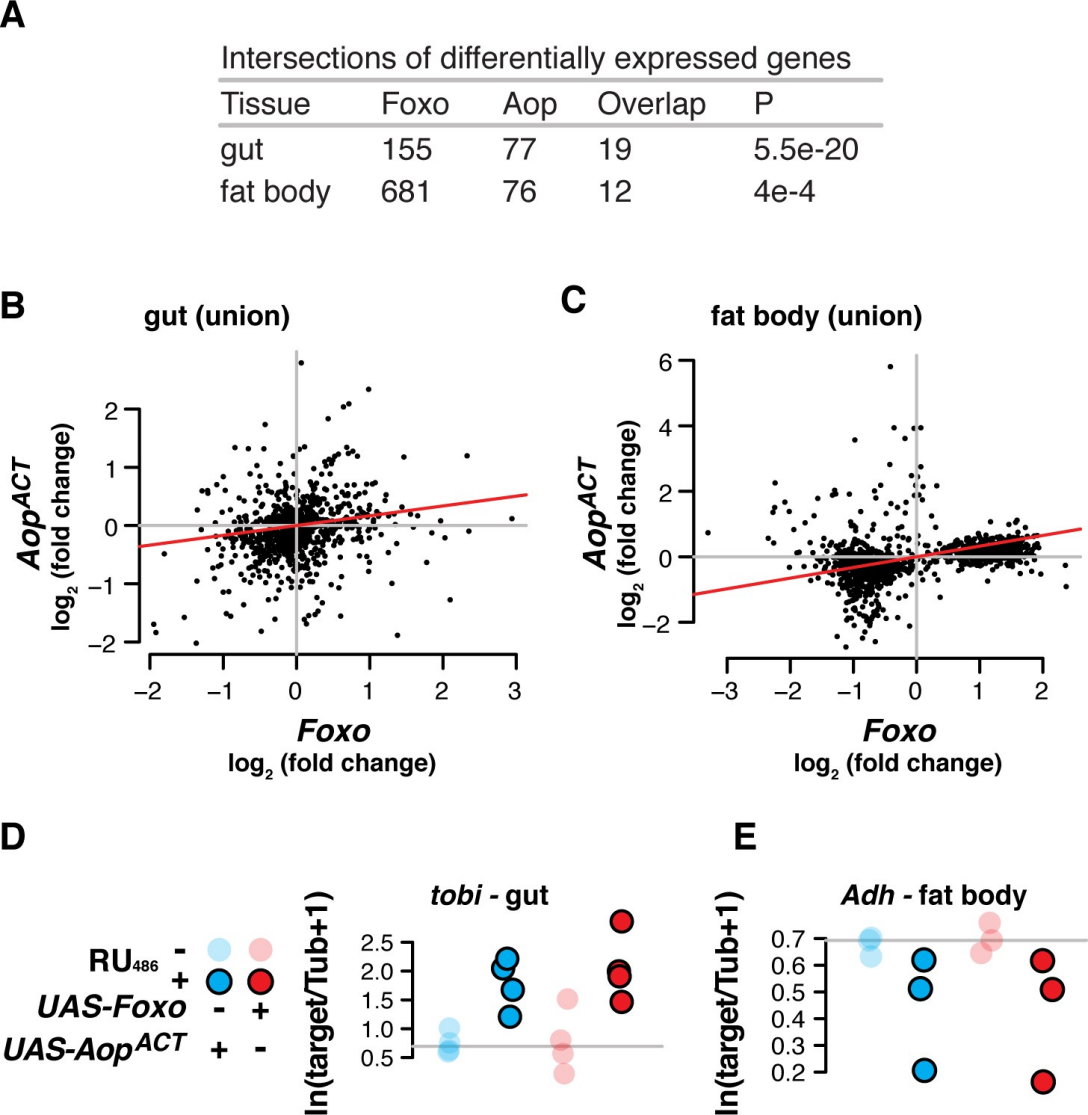
*Aop* recapitulates *Foxo’s* transcriptional output. **(A)** Transcriptomic analysis reveals that overexpression of either *Foxo* or *Aop*^*ACT*^ under control of *S_1_06* induces differential expression of an overlapping set of genes, in both gut and fat body. P values from hypergeometric tests. **(B-C)** In both gut and fat body, in the unions of sets of differentially expressed genes, transcriptomic effects of *Aop*^*ACT*^ correlate those of *Foxo* (log_2_ fold-change expression, calculated by DESeq2). Red lines show correlation coefficients (Kendall’s Tau, p<10e-14 for both tissues). **(D-E)** qRT-PCR confirms congruent effects of *Aop*^*ACT*^ and *Foxo* in fat body and gut (linear model: RU_486_ p<0.05 for either target, no effect of TF).

How does the transcriptional programme triggered by *Aop* relate to that triggered by *Foxo*? We sought to identify genes that were differentially regulated in response to activation of either TF. We focused on adult female fly guts and fat bodies (equivalent to mammalian liver and adipose), since these are the organs from which *Foxo* and *Aop* promote longevity[4]. We induced expression of *Foxo*, *Aop*^*ACT*^ (encoding a constitutively active form of AOP) or both under the control of the *S*_*1*_*106* driver by feeding flies with the RU_486_ inducer. We profiled genome-wide transcriptional changes in dissected guts and abdominal fat bodies (as associated with the cuticle) with RNA-Seq and identified genes responding to RU_486_ within each genotype at a False Discovery Rate (FDR) of 10% (these and all subsequently mentioned gene set assignments are given in S1 Supplementary Information, along with full statistics for all genes in all genotypes; the key to the location of each sheet is contained within the S1 Supplementary Information). In both tissues, we found that the sets of genes regulated by either *Foxo* or *Aop*^*ACT*^ overlapped significantly (gut p<10^−19^, fat body p<10^−4^, Fig 1A). To further assess whether *Aop*’s and *Foxo*’s transcriptional programmes were similar, we tested for correlated expression changes in response to the two TFs within the union of all 712 genes differentially regulated by either TF in the gut, or the equivalent 727 genes in the fat body. The transcriptional programmes triggered by *Foxo* or *Aop*^*ACT*^ were significantly correlated within these unions (Fig 1B and 1C, Kendall's Tau rank-correlation test: gut tau = 0.17, p = 1e-14; fat body tau = 0.32, p<2.2e-16). Interestingly, the sets of differentially expressed genes were largely tissue-specific (S1 Fig), suggesting that this correlated response may be a general feature of the *Aop* and *Foxo* regulons and independent of the tissue-specificity of target promoters. Gene Ontology (GO) enrichment analysis suggested that, in the gut, this combined set of *Aop-* and *Foxo*-regulated genes tended to be involved in translation and energy metabolism, whilst the equivalent analysis in the fat body showed enrichment for regulators of gene expression (details of this GO analysis and all those subsequently mentioned are given in S1 Supplementary Information). We independently confirmed this correlated response to *Aop* and *Foxo* using qRT-PCR of two transcripts identified by transcriptomics: a characterised transcriptional target of IIS [32], *tobi* (Fig 1D, linear model: RU_486_ F_1,13_ = 26.04, p = 2e-4; no effect of genotype, full details of this and all subsequent linear models are contained in one sheet of the S1 Supplementary Information), and alcohol dehydrogenase (*Adh*, Fig 1E*—*linear model: RU_486_ F_1,9_ = 7.83, p = 0.02; no effect of genotype). Hence, *Aop* and *Foxo* not only promote longevity, but also individually effect equivalent transcriptional programmes.

### *Aop* modulates *Foxo*'s transcriptional outputs

**Fig 2:**
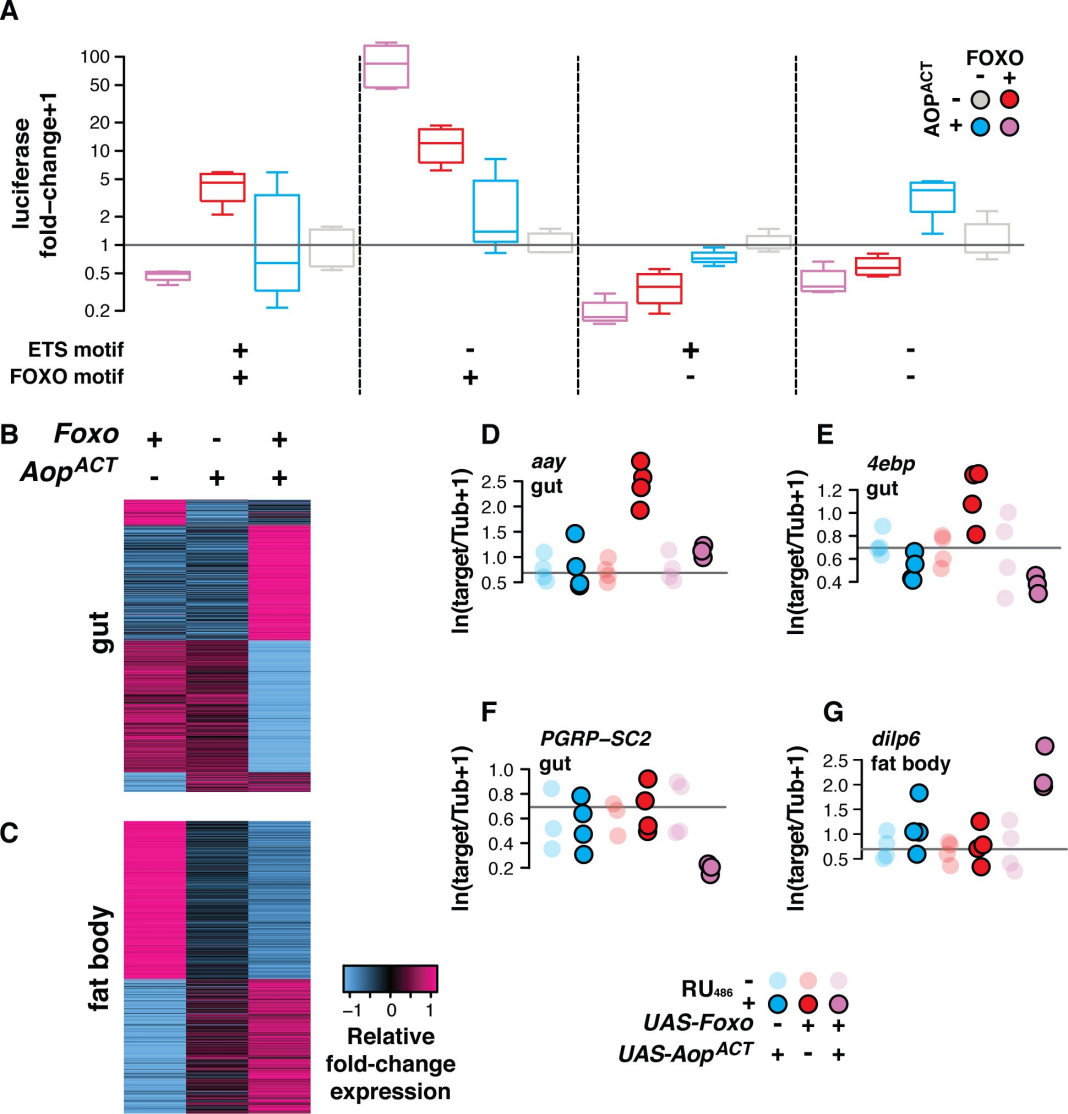
*Aop* modulates *Foxo’s* transcriptional outputs. **(A)** *In vitro* (*Drosophila* S2 cells), AOP^ACT^ alters FOXO's transcriptional output. Representative of three replicate experiments shown, see S2 Fig for each separate replicate and their pool. When both AOP^ACT^ and FOXO can bind a promoter (ETS and FOXO motifs present), AOP^ACT^ directly moderates FOXO activity. When only FOXO can bind a promoter (only FOXO motif present), AOP^ACT^ indirectly synergises with FOXO activity. No significant reporter activity is detected in the absence of FOXO motifs. Statistical modelling confirms that consequence of combining TFs depends on combination of promoter motifs (linear model: Foxo:Aop:FRE:EBM F_1,48_ = 11.41, p = 1e-3). Plot shows luciferase reporter activity from *Drosophila* S2 cells transfected with expression constructs containing combined ETS-binding motifs (EBMs—GGAA) and FOXO-responsive elements (FREs—AACA), upstream of a basal *Adh-Firefly*^*luciferase*^ reporter. Activity is shown following normalisation to internal *Renilla*^*luciferase*^ controls, and calculation of fold-change over the median expression of each reporter in the absence of FOXO and AOP^ACT^. **(B-C)** *In vivo*, expressing *Aop*^*ACT*^ moderates and synergises with *Foxo* to determine transcriptomic output in the gut and fat body (*S_1_06*). Sets of differentially expressed genes regulated interactively by *Aop* and *Foxo* (10% FDR) are shown. Rows represent relative fold-change in expression (Z-scores, log_2_ fold-change expression from DESeq2 output). **(D-G)** qRT-PCR validation of patterns indicated *in vivo* by transcriptomics. In gut, **(D—E)** *Aop*^*ACT*^ abrogates *Foxo's* activation of *aay* and *4ebp* (linear models: *aay* genotype:RU_486_ F_2,17_ = 15.43, p = 1e-4; *4ebp* genotype:RU_486_ F_2,17_ = 8.38, p = 2e-3), while **(F)** they synergise to repress *PGRP-SC2* in the gut (linear model: genotype:RU_486_ F_2,15_ = 4.06, p = 0.03); **(G)** *Aop*^*ACT*^ and *Foxo* synergise to activate *dilp6* in fat body (linear model: genotype:RU_486_ F_2,17_ = 6.61, p = 8e-3).

What are the outcomes of combining *Aop* and *Foxo* activity? FOXO co-localises extensively with AOP in the genome, with 60% of FOXO-bound loci also bound by AOP in the adult gut and fat body[4]. Since AOP functions by repressive interactions with transcriptional activators, we hypothesised that FOXO activity would be modulated by AOP. We tested this hypothesis *in vitro*. Transcriptional reporters were constructed by combining the *Adh* basal promoter with FOXO-responsive elements (FREs: AACA), ETS-binding motifs (EBMs: GGAA) or both, and examined for their response to FOXO and AOP^ACT^ in *Drosophila* S2 cells (Fig 2A, S2 Fig). In the presence of EBMs, AOP prevents activation by ETS activators [e.g. 33]. In the presence of FREs, FOXO is known to activate transcription [31]. We confirmed published observations for individual TFs on the reporters that contained their individual binding elements: FOXO was sufficient to activate transcription from the FREs (t-test t = 6.64, p = 3.7e-5), while, as expected [23,26,28], AOP^ACT^ did not impact expression from EBMs (t-test t = -0.66, p = 0.26).

We conducted three replicate experiments to assess the interactive output of AOP and FOXO. Combining the FREs and EBMs allowed AOP^ACT^ to attenuate activation by FOXO, revealing that AOP can moderate FOXO’s activity when brought onto the same promoter. By striking contrast, in the absence of EBMs, AOP^ACT^ synergised with FOXO to stimulate induction to an order of magnitude greater than FOXO alone, indicating that AOP^ACT^ can indirectly accentuate FOXO’s ability to activate transcription (Fig 2A). While the magnitude of these effects varied, it was consistently present across three independent experiments (S2 Fig). To analyse these data we used linear modelling, testing how the complement of TF binding motifs altered the output of combining the TFs, both in each individual experiment, and across the three experiments. This analysis confirmed that the output of combining AOP and FOXO was promoter-dependent (Linear model: FOXO:AOP:FRE:EBM—data from all three replicates, F_1,158_ = 21.06, p = 9e-6; data from Fig 2A F_1,48_ = 15.34, p = 2e-4; see also statistical analysis section of S1 Supplementary Information). Since the synergistic interaction occurred in the absence of EBMs, this is most likely an indirect effect, occurring not via a direct interaction on the promoter but rather via AOP-induced transcriptional changes elsewhere in the genome. Note that in the presence of EBMs, any synergistic effect of AOP appears counteracted by the repression occurring from direct AOP binding to the promoter. Synergy may account in part for the similarity of AOP’s and FOXO’s transcriptional programmes *in vivo* (Fig 1). Hence, AOP is not only able to moderate the activity of other ETS activators, but also the Forkhead TF FOXO, with the presence or absence of EBMs in a promoter determining whether AOP enhances or moderates FOXO activity.

The *in vitro* analysis suggested that *Foxo*’s *in vivo* output should depend on *Aop* activity. To examine if synergy and antagonism of *Foxo* by *Aop* can be observed on native promoters *in vivo*, we looked at what happens when *Aop* and *Foxo* were combined. We used our above-described RNA-Seq experiment and sorted the union of differentially expressed genes by the direction of regulation upon induction of *Foxo*, *Aop*^*ACT*^, or both, paying attention to altered regulation when the TFs were co-induced. To visualise the groupings, we compared the fold-change values for each gene between different conditions by calculating per gene Z-score (number of standard deviations away from the mean fold-chage; Fig 2B and 2C). In this way, we could identify sets of genes that may be synergistically or antagonistically regulated by *Aop* and *Foxo*. We note that neither *Aop* nor *Foxo* were significantly down-regulated by the other in either tissue, indicating that their combined transcriptomic outputs result from interactive effects on promoters (S1 Supplementary Information). We selected specific candidates for validation by qRT-PCR, and used linear models to test for interactive effects of the TFs, indicated by differential effects of RU_486_ feeding on the study genotypes. Indeed, we found that *Aop* was able to antagonise *Foxo*’s induction of *aay* and *4ebp* in the gut (Fig 2D and 2E; *aay* genotype:RU_486_ F_2,17_ = 15.43, p = 1e-4; *4ebp* genotype:RU_486_ F_2,17_ = 8.38, p = 2e-3; full analysis in S1 Supplementary Information). On the other hand, *Aop* synergised with *Foxo* to modulate expression of *PGRP-SC2* in the gut and *dilp6* in the fat body (Fig 2F and 2G; *PGRP-SC2* genotype:RU_486_ F_2,15_ = 4.06, p = 0.03; *dilp6* genotype:RU_486_ F_2,17_ = 6.61, p = 8e-3; full analysis in S1 Supplementary Information). Thus, transcript profiling followed by qRT-PCR validation confirmed that the two modes of AOP:FOXO interaction observed on synthetic reporters can also occur *in vivo*. This simultaneous synergy and antagonism of AOP and FOXO may explain why, whilst activation of either TF is sufficient to promote longevity, their co-activation does not extend lifespan additively[4].

### *Aop* and *Foxo* broker transcriptomic outcomes *in vivo* with *Pnt*

**Fig 3:**
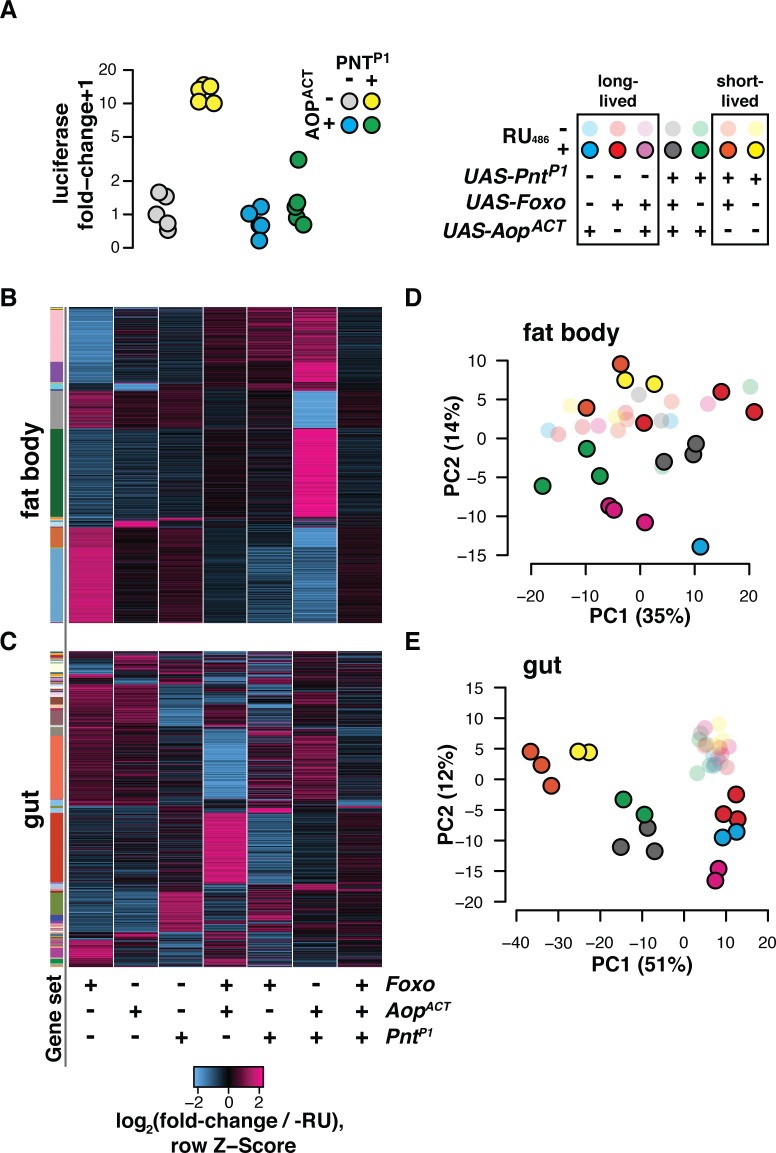
*Aop* and *Foxo* broker transcriptomic outcomes with *Pnt*. **(A)** *In vitro* (*Drosophila* S2 cells) AOP^ACT^ counteracts activation by PNT^P1^ of a synthetic promoter containing EBMs, upstream of an *Adh-Firefly*^*luciferase*^ reporter. Activity is shown following normalisation to internal *Renilla*^*luciferase*^ controls, and calculation of fold-change over median expression in the absence of PNT^P1^ and AOP^ACT^. Promoter activation was subject to a significant AOP^ACT^:PNT^P1^ interaction (linear model F_1,16_ = 41.725, p = 7.9e-6) **(B-C)** *Foxo* and *Aop*^*ACT*^ coordinate transcription by jointly countering *Pnt*'s transcriptional output. Heatmaps show per-gene Z-score of log_2_ fold-change expression (DESeq2 output) upon RU_486_ feeding relative to controls. Rows represent all genes in the union of targets identified in flies bearing *S_1_06* transgenes along with *UAS-Foxo*, *UAS-Aop*^*ACT*^, *UAS-Pnt*^*P1*^, or combinations thereof. Gene sets are defined by pattern of response to RU_486_ amongst genotypes. Set assignments shown by coloured side bar. **(D-E)** *Aop*^*ACT*^, *Foxo* and *Pnt*^*P1*^ interact to establish transcriptional programs corresponding to lifespan. For gut and fat body, plots show coordinates of samples on the first two dimensions of PCA for the genes whose expression was responsive to the combinatorial, interactive effects of the three TFs. Key shows samples’ groupings by previously-published lifespan outcomes[4] resulting from TF induction in the gut and fat body or the gut alone (noting that lifespan effects of combined *Aop*^*ACT*^ and *Pnt*^*P1*^ expression are not known).

Whilst interactions with FOXO appear to account for some of the transcriptional outputs of AOP, 80% of AOP-bound genomic sites are not bound by FOXO *in vivo*[4]. Since AOP alone is insufficient to regulate transcription when brought onto a promoter (Fig 2A and references[21,23,26,28]), interactions with other transcriptional activators must account for the full breadth of *Aop*'s physiological and transcriptomic effects. *Pnt* is one such transcriptional activator. *Pnt* and *Aop* have mutually antagonistic roles in development, which is presumed to occur by competition for binding sites since the two recognise the same DNA sequence[23,30,34]. We confirmed this interaction on reporters in S2 cells: Transcriptional induction by PNT^P1^ (a constitutively active isoform[35]) was completely blocked by AOP^ACT^ (Fig 3A, linear model AOP:PNT F_1,16_ = 41.8, p = 7.9e-6; also see references[23,28,36,37]), suggesting that PNT inhibition may be a key factor in *Aop*’s pro-longevity effect. Additionally, *Pnt* over-expression can block the longevity effects of both *Foxo* and IIS [4,9], suggesting that *Pnt* may also modify *Foxo’s* transcriptional output. To evaluate emergent interactions *in vivo*, the transcriptome-wide effects of co-expressing *Aop*^*ACT*^, *Pnt*^*P1*^ *and Foxo* in the gut and fat body were examined.

We assessed the transcriptomic outcomes of induction of *Pnt*^*P1*^ either alone or in combination with *Aop*^*ACT*^ and *Foxo* (note that this is an extension of the above-described transcriptomic experiment, which was performed at the same time). For each of the gut and the fat body, we assembled sets of genes that were differentially regulated upon induction of any of the three TFs or their combinations (union of all genes differentially expressed at FDR 10%, set assignments per tissue in S1 Supplementary Information, noting that the preceding Foxo/Aop-regulated genes are a subset). This formed a union of 945 genes in the gut, and 1214 genes in the fat body. We sorted these genes by their pattern of regulation (i.e. set assignment) and visualised the groupings based on per-gene Z-score. This revealed a complex pattern in both tissues where each TF appeared able to influence the outcomes of the other two (Fig 3B and 3C). To distil these interactions, we tested explicitly for genes whose regulation is subject to a statistically significant three-way interaction of *Foxo*, *Aop*^*ACT*^ and *Pnt*^*P1*^ induction. In the gut, 511 transcripts were subject to the combinatorial, interactive effects of the three TFs, as were 617 in the fat body (10% FDR, see results in S1 Supplementary Information). To reveal emergent transcriptional programmes in each tissue, principal components analysis (PCA) was performed over these sets of transcripts (Fig 3D and 3E). Remarkably, the first principal component (PC) of differentially expressed genes in the gut distinguished flies by published lifespan outcomes[4], with short-lived flies expressing *Pnt*^*P1*^ alone or in combination with *Foxo* at one end of the PC; long-lived flies expressing one or both *Foxo* and *Aop*^*ACT*^ forming a distinct group at the other end of the PC; and *Aop*^*ACT*^ countering the effect of *Pnt*^*P1*^ to form an intermediate group (Fig 3E). In the fat body, a similar grouping was apparent on the diagonal of PCs 1 and 2 (Fig 3D), despite more variability in the data, probably resulting from the difficulty of dissecting this organ. To infer functional consequences of these distinct transcriptional programmes, transcripts from the input set corresponding to the PCs were isolated and GO enrichment analysis performed. This revealed a strong enrichment of genes with roles in energy metabolism, whose expression was strongly correlated to the PCs (S3 Fig). Overall, a combined view of the PCA and GO analysis predicted that: (1) inhibiting *Pnt* may recapitulate the transcriptional programme of *Aop* and *Foxo* and promote longevity, and (2) that *Pnt*, alongside *Aop* and *Foxo*, may regulate metabolism in young flies.

### *Pnt* limits lifespan

**Fig 4:**
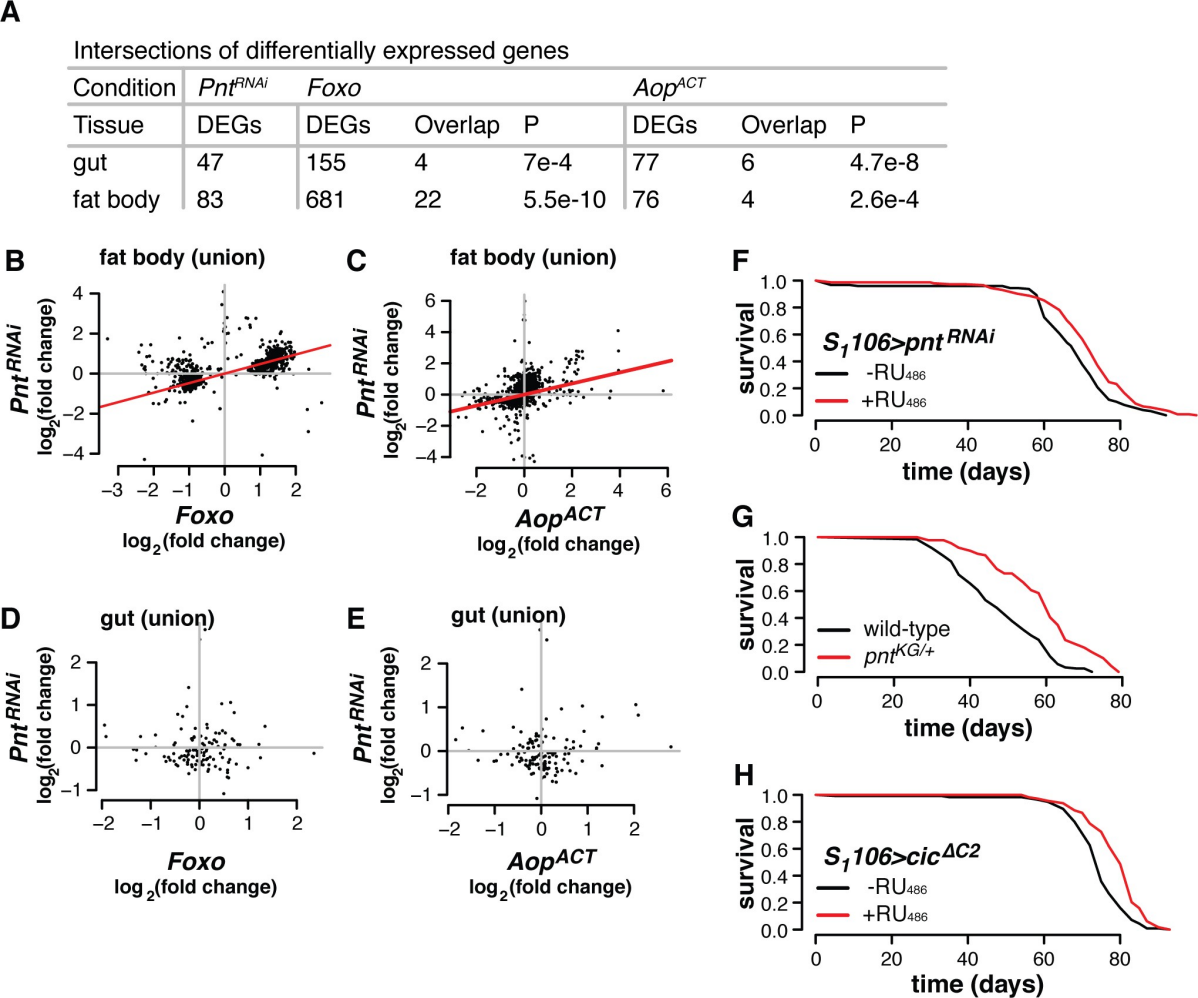
Physiological *Pnt* regulates lifespan and genes in the *Aop/Foxo* regulon. **(A)** Transcriptomic analysis reveals that expressing RNAi against *Pnt* under control of *S_1_06* induces differential expression of set of genes that overlap with the *Foxo-Aop* regulon, in both gut and fat body. P-values from hypergeometric tests. **(B-C)** In fat body, in the union of transcripts responding to *Pnt*^*RNAi*^, *Foxo* or *Aop*^*ACT*^, impacts of *Pnt*^*RNAi*^ are correlated to those of *Foxo* or *Aop*^*ACT*^ (log_2_ fold-change expression from DESeq2 output). Red lines show correlation coefficients (Kendall’s Tau, P≤2.2e-16 for both tissues). **(D-E)**. In gut, in the union of transcripts responding to *Pnt*^*RNAi*^, *Foxo* or *Aop*^*ACT*^, impacts of *Pnt*^*RNAi*^ do not correlate those of *Foxo* or *Aop*^*ACT*^ (log_2_ fold-change expression from DESeq2 output). **(F)** Adult-onset *Pnt* inhibition in the gut and fat body is sufficient to extend lifespan. Log-rank test p = 7.2e-4. **(G)** Heterozygous *Pnt* mutants are long-lived. Log-rank test p = 9.2e-11. **(H)** Overexpressing *Cic* (an inhibitor of *Pnt*), in the gut and fat body extends lifespan. Log-rank test p = 1.5e-7.

Since *Aop* and *Foxo* appeared to drive a transcriptional programme opposed to that of *Pnt*, we hypothesised that directly limiting physiological levels of *Pnt* would be sufficient to recapitulate their effect on gene expression. We first assessed the transcriptome-wide changes in the gut and fat body induced by RNAi-mediated knockdown of *Pnt*. The sets of genes differentially regulated by *Pnt* knockdown (FDR 10%) significantly overlapped those regulated by *Aop*^*ACT*^ or *Foxo* in both the gut and the fat body (Fig 4A). Additionally, in the union of the genes regulated by *Pnt*^*RNAi*^, *Aop*^*ACT*^ or *Foxo* induction in the fat body, correlated effects of *Pnt* knockdown and *Foxo* or *Aop* activation were evident (Fig 4B and 4C). However, such broad correlations were not evident in the gut (Fig 4D and 4E). Hence, reducing the physiological levels of *Pnt* can recapitulate some aspects of the *Aop/Foxo* transcriptional programme. But is this sufficient to extend lifespan?

Inducing RNAi against *Pnt* from day three of adulthood in the gut and fat body was indeed sufficient to increase lifespan (Fig 4F, log-rank p = 7.2e-4). To further validate this finding, we backcrossed a loss-of-function p-element insertion in *Pnt* (*Pnt*^*KG04968*^, henceforth *Pnt*^*KG*^), into an outbred, wild-type background for ten generations. The mutation was homozygous lethal. However, heterozygote females exhibited a 20% increase in median lifespan (Fig 4G, log-rank p = 9.2e-11). We also tested whether expressing a transcriptional repressor of the *Pnt* locus extended lifespan. The HMG-box repressor *capicua* (*cic*) represses expression of *Pnt*[38] and, consistent with the effects of *Pnt*^*RNAi*^, overexpressing *cic*^*ΔC2*^ (a *cic* mutant lacking a known MAPK phosphorylation site) in the gut and fat body also substantially extended lifespan (Fig 4H, log-rank p = 1.5e-7). These experiments demonstrated that countering *Pnt* is sufficient to recapitulate aspects of the *Aop*/*Foxo* transcriptional programme and extend lifespan, and corroborate the conclusions of transcriptomic analysis that *Aop* and *Foxo* act in part by countering *Pnt*.

### *Pnt* determines metabolic outcomes

**Fig 5:**
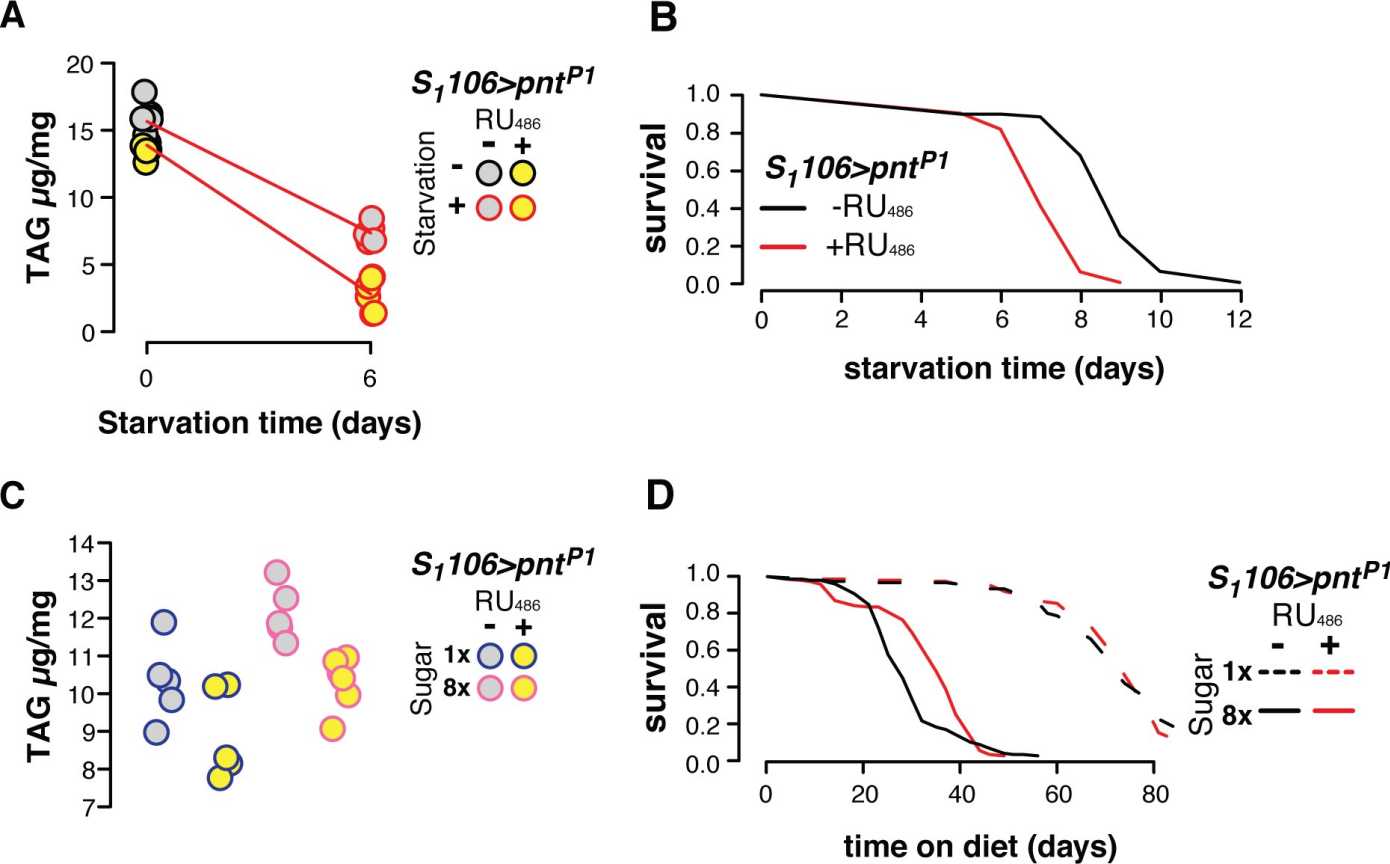
*Pnt* impacts the response to nutritional stress. **(A)** Over-expressing *Pnt*^*P1*^ in the gut and fat body accelerates loss of TAG under starvation stress (linear model: RU_486_:starvation F_1,19_ = 7.03, p = 0.02; see S4 Fig for corresponding plot of body mass). **(B)** Over-expressing *Pnt*^*P1*^ in the gut and fat body reduces survival under starvation stress (log-rank test p = 1.3e-14). **(C)** Over-expressing *Pnt*^*P1*^ in the gut and fat body reduces accumulation of TAG on a high-sugar diet. *Pnt*^*P1*^ reduced TAG (linear model F_1,17_ = 14.4, p = 1.4e-3), and sugar increased TAG (linear model: F_1,17_ = 15.25, p = 1.1e-3), with *Pnt*^*P1*^ appearing to counteract the effect of sugar (mean±SE: low sugar-RU_486_ 10.31±0.48, high sugar+RU_486_ 10.30±0.29; t-test t = 0.009, p = 0.99). **(D)** Over-expressing *Pnt*^*P1*^ in the gut and fat body enhances survival on a high-sugar diet (Cox Proportional Hazards diet:RU_486_ p = 6e-3).

Our data show that each member of the *Foxo-Aop-Pnt* circuit can be targeted in the gut and fat body to extend lifespan. What is the function of this circuit, and *Pnt* in particular, in young flies, before ageing occurs? The RNA-Seq data sets suggested metabolic regulation. Since the levels of *Pnt* appeared to dictate transcriptional and lifespan outcomes (Figs 3 and 4), we evaluated its metabolic role in more detail. The presence of genes including lipases and perilipin (*Lsd-2*) in the transcriptome data suggested that *Pnt* modulates lipid metabolism. Therefore, we applied nutritional stresses to alter triacylglyceride (TAG) storage, and assessed how *Pnt*^*P1*^ altered the response to these stresses. We quantified TAG after a week of *Pnt*^*P1*^ induction, and then after a subsequent six days of starvation. *Pnt*^*P1*^ accentuated the loss of TAG per unit weight induced by starvation (Fig 5A; linear model RU_486_:starvation F_1,19_ = 7.03, p = 0.02), but not overall weight loss (S4 Fig), suggesting that *Pnt* sensitises flies specifically to cues for lipolysis. The mobilisation of TAG stores was associated with decreased resistance to starvation, with flies over-expressing *Pnt*^*P1*^ dying 24% earlier on average (Fig 5B log-rank p = 1.3e-14). This ability of *Pnt* to promote catabolism of energy stores may be beneficial in the face of over-nutrition, and relevant to the Western human epidemic of metabolic disease associated with energy-rich diets. A *Drosophila* model of such energy-rich diets is feeding flies a high sugar diet. Flies fed 40% sugar die substantially earlier than controls fed a 5% sugar diet, and accumulate TAG[39,40]. However *Pnt*^*P1*^ overexpression restored TAG levels in flies on a high-sugar diet to those observed on a low-sugar diet (Fig 5C). Whilst there was no statistically significant interaction of sugar and *Pnt*^*P1*^ induction in a linear model (RU_486_:sugar F_1,17_ = 0.32, p = 0.57), the adipogenic effect of sugar was opposed by *Pnt*, such that TAG levels on a high-sugar diet with *Pnt* induction were not different from those on a low-sugar diet without *Pnt* induction (t-test: t = 0.01, p = 0.99). Moreover, *Pnt*^*P1*^ induction spared flies from the full extent of the early death induced by dietary sugar, increasing median survival time by 26%, despite having no effect on the low-sugar diet (Fig 5D; cox proportional hazards RU_486_:sugar p = 6.2e-3). Altogether, these results indicate that while *Pnt* activity is detrimental during ageing, in youth it predisposes flies to leanness, which correlates survival of nutritional stress. This may suggest that metabolic regulation is an adaptive function of the *Foxo-Aop-Pnt* circuit in early life, but that the configuration which is optimal for metabolism is deleterious for later survival.

### The majority of ETS TFs limit fly lifespan

**Fig 6:**
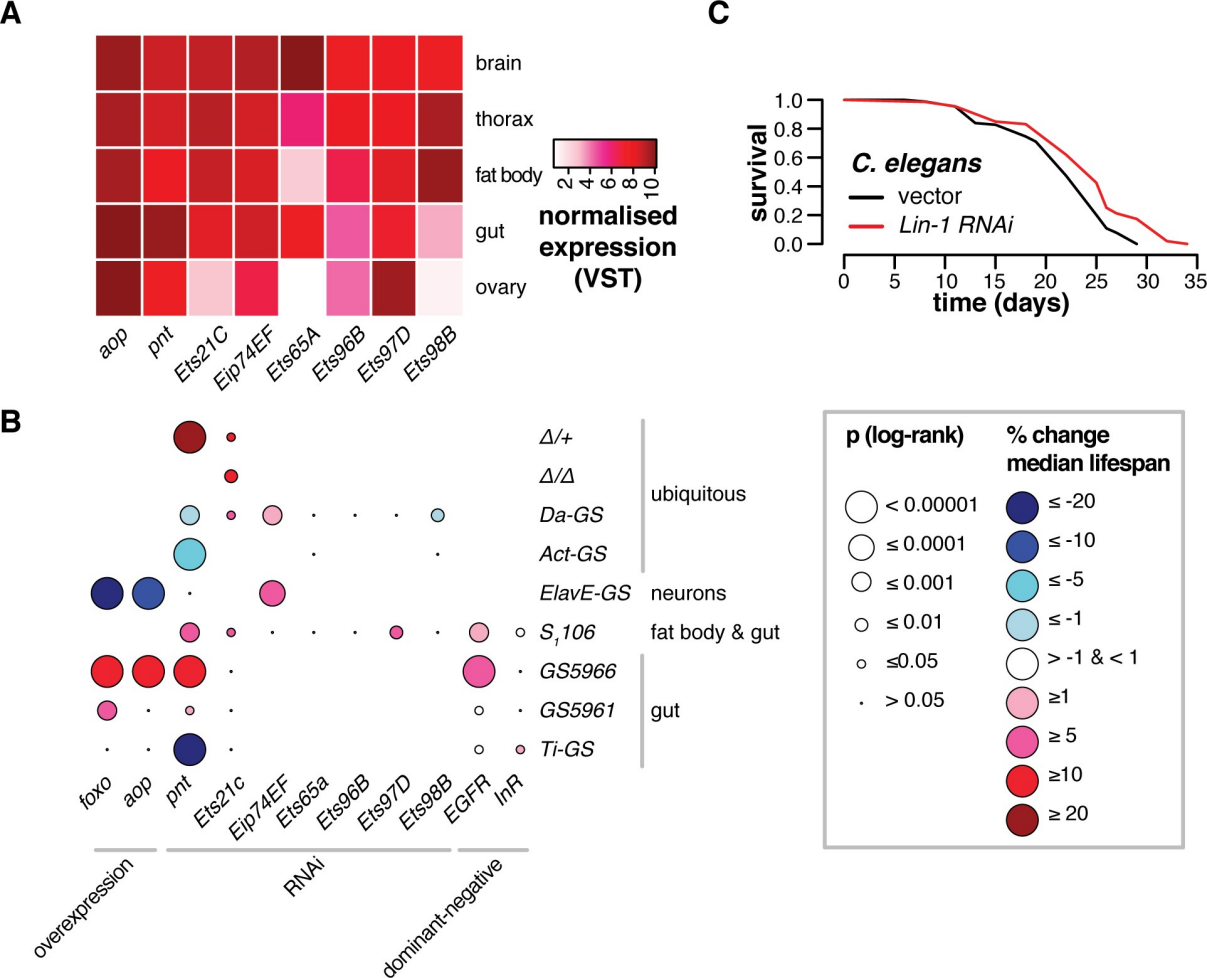
Lifespan regulation is a conserved function of ETS TFs. **(A)** The diversity and tissue-specific expression of ETS TFs in *Drosophila*. A variance-stabilising transformation was applied to published expression data from the same population of flies and same media as used in Figs 1–5 [5], and medians were calculated. **(B)** Tissue-specific lifespan regulation is a conserved function of ETS TFs in *Drosophila*. Plot summarises 44 lifespan experiments. Bubbles are colour-coded for percentage change in median lifespan, either in mutants relative to controls, or upon feeding RU_486_ to flies bearing the indicated geneswitch drivers along with either RNAi or overexpression constructs. Bubble size represents p-values from log-rank tests. Full genotype information and p-values are given in S1 Supplementary Information. Note that lifespans of *S_1_06* flies overexpressing dominant-negative *InR* were significantly extended due to delayed mortality in early or late portions of survival curves, despite absence of differential median survival (See S8 Fig). **(C)** ETS TFs have evolutionarily-conserved functions in lifespan: Feeding *lin-1*^*RNAi*^ to the nematode *C*. *elegans* from egg promotes longevity (log-rank test p = 3.3e-2).

Animal genomes encode multiple ETS factors: In *Drosophila* the ETS family comprises the repressor *Aop* and activators: *Pnt*, *Eip74EF*, *Ets21C*, *Ets65A*, *Ets96B*, *Ets97D and Ets98B*, each of which is expressed with its own unique tissue-specific pattern (Fig 6A). Finding lifespan-limiting roles of *Pnt* in addition to the previously described pro-longevity role of *Aop*, suggested that other ETS TFs with functions as transcriptional activators may also have the same lifespan-limiting effect. We examined the function of the other ETS TFs in *Drosophila* lifespan by knocking down their expression levels with RNAi in combination with inducible drivers. The data obtained in >40 lifespan assays are summarised in Fig 6B, including information on *Aop*, *Pnt* and *Foxo*. Summary statistics of each lifespan, along with associated genetic information, are presented in S1 Supplementary Information, while individual lifespan curves are presented in S5–S8 Figs. We identified each of *Eip74EF*, *Ets21C* and *Ets97D* as limiting lifespan in at least one tissue. For *Ets21C*, we confirmed the result using an available mutant (S5 Fig). Whilst some of the effects we observed were modest, overall the data pointed to roles in ageing for five of the eight *Drosophila* ETS TFs.

The effects were in general tissue specific. RNAi against *Pnt*, *Ets21C* and *Ets97D* restricted to the gut and the fat body with the *S*_*1*_*106* driver extended lifespan (Fig 6B, S5 Fig), the same tissues where *Foxo* and *Aop* act [4]. Knockdown of *Pnt* but not of *Ets21C* in enterocytes (ECs), using the *GS5966* driver, was sufficient to extend lifespan, as was activating either *Aop*^*ACT*^ or *Foxo* (Fig 6B, S6 Fig). *Pnt* and *Ets21C* have been characterised as regulators of intestinal stem cell (ISC) proliferation[38], but activating cognate RNAi constructs with drivers that are active in ISCs (GS5961 and TIGS) did not consistently or substantially extend lifespan (Fig 6B, S6 Fig). *Pnt* functions in neurogenesis[41], and its continued expression in adult neurons suggested an ongoing, physiologically relevant role. However, neuronal *Pnt*^*RNAi*^ induction, achieved with the *ElavGS* driver, did not affect lifespan, while over-expressing either *Aop*^*ACT*^ or *Foxo* was deleterious and in contrast to their benefits in gut and fat body (Fig 6B, S7 Fig). *Eip74EF* is more highly expressed in the brain than other tissues (Fig 6A), indicating that neurons may mediate the beneficial effect of its ubiquitous knockdown. Indeed, expressing *Eip74EF*^*RNAi*^ in neurons using the inducible, neuron-specific driver *Elav-GS* extended lifespan (Fig 6B, S7 Fig). Overall, these data show that members of the *Drosophila* ETS family, along with *Foxo*, have distinct effects on lifespan in distinct tissues.

The ETS TFs act downstream of receptor tyrosine kinase (RTK) pathways. The insulin receptor *InR* is an established regulator of *Aop* and *Foxo*[8], and reducing its activity promotes lifespan[9]. Whilst expressing *InR*^*DN*^ (a dominant-negative form) in the gut and fat body enhanced lifespan, expressing the same construct in ECs did not (Fig 6B, S8 Fig), indicating that another RTK may function upstream of ETS TFs and *Foxo* in the ECs. The epidermal growth factor receptor *EGFR* can signal to *Pnt* and *Ets21C* via cic[38], suggesting *EGFR* in ECs may regulate lifespan. Indeed, inducing the dominant-negative form *EGFR*^*DN*^ in the gut and fat body or ECs extended lifespan (Fig 6B, S8 Fig). Hence, different ETS factors may limit lifespan downstream of different RTK pathways in different tissues.

The evidence suggested that a role in ageing is shared amongst multiple ETS factors in *Drosophila*. ETS TFs are conserved throughout multicellular animals, and the extensive conservation of roles in lifespan amongst the ETS family in the fly suggested that this lifespan modulation may be a fundamental property of these TFs, that extends to other species. The genome of the nematode *C*. *elegans* encodes 11 ETS TFs in total. At least one of these, *Ets-4*, has been reported to limit lifespan in the worm intestine[17]. We screened the majority of the other *C*. *elegans* ETS TFs for roles in lifespan by feeding worms RNAi from egg or L4 onwards (S1 Supplementary Information). Expanding the repertoire of proteins that limit worm lifespan, we found that knockdown of *Lin-1* (an orthologue of human *ELK1*, *ELK3* and *ELK4*) consistently extended *C*. *elegans* lifespan, in multiple independent trials from L4 stage or egg (e.g. Fig 6C). Thus, multiple ETS factors limit lifespan in species separated by hundreds of millions of years of evolutionary divergence, hinting at a general role for this family of TFs in animal longevity.

## Discussion

Promoting healthy ageing by transcriptional control is an attractive prospect, because targeting one specific protein can restructure global gene expression to provide broad-scale benefits. This study suggests key roles for ETS TFs in such optimisation. The results show dual roles for *Aop*: balancing *Foxo*’s outputs, and opposing *Pnt*’s outputs. These functions coordinate transcriptional changes that correspond to lifespan. Repressing transcription from the ETS site appears to be the key longevity-promoting step, and indeed lifespan was extended by limiting multiple ETS TFs, in multiple fly tissues, and in multiple taxa. Altogether, these results show that inhibiting lifespan is a general feature of ETS transcriptional activators. Presumably the expression of these TFs is maintained, despite costs in late life, because of benefits in other contexts. For example, *Pnt* is important during development[23,34–36], and expression may simply run-on into adulthood. We have now shown that *Pnt* is also important for adults facing nutritional variation or stress, and genomic evidence suggests equivalent functions for *Ets-4* in *C*. *elegans*[17]. In addition, *Ets21C* is required to mount an effective immune response[42], and both *Ets21C* and *Pnt* control gut homeostasis[38]. Tissue environment appears to be another important contextual factor that determines the lifespan effects of specific ETS TFs. Differences between tissues in chromatin architecture are likely to alter the capacity of a given TF to bind a given site, and our results show that a given TF, and also upstream RTKs, do not necessarily lead to the same lifespan effect across all tissues. The tissue-specific functions that we show for ETS TFs, *Foxo* and RTKs, suggests that transcription is locally coordinated by distinct receptors and TFs in distinct tissues, but that lifespan-regulatory signalling nevertheless converges on the ETS site. This differentiation makes it all the more remarkable that roles in lifespan appear to be conserved amongst ETS family TFs, even in diverse tissue contexts.

The structure of molecular networks and their integration amongst tissues underpins phenotype, including into old age. Unravelling the basics of these networks is a critical step in identifying precise anti-ageing molecular targets. Identifying the least disruptive perturbation of these networks, by targeting the “correct” effector, is a key goal in order to achieve desirable outcomes without undesirable trade-offs that may ensue from broader-scale perturbation. This targeting can be at the level of specific proteins, cell types, points in the life-course, or a combination of all three. The tissue-specific expression pattern of ETS TFs, and the apparent conservation of their roles in longevity, highlights them as important regulators of tissue-specific programs that may be useful in precise medical targeting of specific senescent pathologies.

## Methods

### *D*. *melanogaster* culture

All experiments were carried out in outbred, *Wolbachia*-free *Dahomey* flies, bearing the *w^1118^* mutation and maintained at large population size since domestication in 1970. All transgenes (S1 Supplementary Information) were backcrossed into this background at least 6 times prior to experimentation, and stocks were maintained without bottlenecking. Cultures were maintained on 10% yeast (MP Biomedicals, OH, USA), 5% sucrose (Tate & Lyle, UK), 1.5% agar (Sigma-Aldrich, Dorset, UK), 3% nipagin (Chemlink Specialities, Dorset, UK), and 0.3% propionic acid (Sigma-Aldrich, Dorset, UK), at a constant 25°C and 60% humidity, on a 12:12 light cycle. Experimental flies were collected as embryos following 18h egg laying on grape juice agar, cultured at standardised density until adulthood, and allowed to mate for 48h before males were discarded and females assigned to experimental treatments at a density of 15 females/vial. To induce transgene expression using the GeneSwitch system, the inducer RU_486_ (Sigma M8046) was dissolved in absolute ethanol and added to the base medium to a final concentration of 200 μM. Ethanol was added as a vehicle control in RU-negative food. For lifespan experiments, flies were transferred to fresh food and survival was scored thrice weekly. Feeding RU_486_ to driver-only controls did not affect lifespan (S1 Supplementary Information). For starvation stress experiments, flies were fed RU_486_ or EtOH-supplemented media for one week, before switching to 1% agarose with the equivalent addition of RU_486_ or EtOH, with death scored daily until the end. For sugar stress experiments, sugar content was increased to 40% w/v sucrose[39,40].

### *C*. *elegans* culture

Worms were maintained by Brenner’s protocol[43], at 20°C on NGM plates seeded with *Escherichia coli* OP50. For lifespan experiments, N2 (wildtype N2 male stock, N2 CGCM) were used at 20°C on NGM plates supplemented with 15μM FUDR to block progeny production. RNAi treatment was started from egg or late larval stages (details in Supplementary Materials). Animals that died from internal hatching were censored.

### Molecular cloning

The *pGL3Basic-4xFRE-pADH-Luc* construct (called pGL4xFRE, reference [31]) was used as template to generate PCR products containing 6xETS-4xFRE-pADH, 4xFRE-pADH, 6xETS-pADH- or pADH (primers in S1 Supplementary Information, ETS sequence described in [44]), flanked by *XhoI* and *HindIII* sites, cloned into the corresponding sites in *pGL3-Basic* and confirmed by sequencing. *PntP1* was amplified from *UAS-Pnt*^*P1*^ genomic DNA with Q5 High-Fidelity Polymerase (NEB M0491S - primers in S1 Supplementary Information), and *Aop*^*ACT*^ was cloned from genomic DNA of *UAS-Aop*^*ACT*^ flies[4]. *Pnt*^*P1*^ and *Aop*^*ACT*^ sequences were then cloned into the *pENTR-D-TOPO* gateway vector (Thermo 450218) before recombination into the *pAW* expression vector.

### S2 cell culture

*Drosophila* S2 cells were cultured in Schneider’s medium (Gibco/Thermo Scientific 21720024), supplemented with 10% FBS (Gibco/Thermo Scientific A3160801) and Penicillin/Streptomycin (Thermo 15070063). Cells were split into fresh media 24h before transfection, then resuspended to a density of 10^6^ ml^-1^ and transfected using Effectene reagent (Qiagen 301425) in 96-well plates, according to the manufacturer’s instructions. Reporters and TF expression plasmids were co-transfected with *pAFW-eGFP* to visually confirm transfection, and *pRL-TK-Renilla*^*luc*^ as an internal control for normalisation of reporter-produced Firefly luciferase. Reporters and *pRL-TK-Renilla*^*luc*^ were transfected 1:1. When multiple TF expression plasmids were transfected, it was done 1:1. Each TF expression plasmid was transfected 4:1 relative to reporters or *pRL-TK-Renilla*^*luc*^ (i.e. for every ng TF expression plasmid, 0.25 ng reporter and 0.25 ng *pRL-TK-Renilla*^*luc*^ were transfected). The total amount of DNA transfected was then topped up to a standard quantity across all experimental conditions with *pAFW-eGFP*, in equal volumes of TE buffer. Reporter activity was measured 18h after transfection using Dual-Luciferase reagents (Promega E1960). *pAHW-Foxo* and/or *pAW-Aop*^*ACT*^ were co-transfected with promoters bearing combinations of FREs and EBMs. *pAW-Aop*^*ACT*^ and *pAW-Pnt*^*P1*^ were co-transfected with a promoter bearing EBMs.

### Transcriptomics

Flies bearing combinations of *UAS-Foxo*, *UAS-Aop*^*ACT*^ and *UAS-PntP1*, or *UAS-Pnt*^*RNAi*^ in an *S_1_06-GS* background were dissected after six days adult feeding on RU_486_. Tissues were dissected in ice-cold PBS. Guts were dissected by cutting off the head and last abdominal segment, pulling on the crop through an incision at the abdomenal-thoracic junction, then removing tubules. Reproductive anatomy was then removed from the abdomen and the remainder of the abdomen taken as fat body. Dissected tissues were placed directly into ice-cold Trizol (Ambion 15596026). In the *Foxo-Aop*^*ACT*^*-Pnt*^*P1*^ epistasis RNA-Seq experiment, four experimental replicates were sampled per condition, each comprising a pool of 12 fat bodies or guts. In the *Pnt*^*RNAi*^ experiment three replicates were sampled per condition, also each comprising organs from 12 flies. RNA was extracted by Trizol-chloroform extraction, quantified on a NanoDrop, and integrity was assessed on an Agilent Bioanalyzer. Poly(A) RNA was pulled down using NextFlex Poly(A) beads (PerkinElmer NOVA-512981) and integrity was re-assessed. In the *Foxo-Aop*^*ACT*^*-Pnt*^*P1*^ epistasis RNA-Seq experiment, only samples with the highest RNA yields and integrity were included in library preparation, leaving 2–3 samples per experimental condition. All three replicates were prepped and sequenced in the *Pnt*^*RNAi*^ RNA-Seq experiment. RNA fragments were given unique molecular identifiers and libraries were prepared for sequencing using NextFlex qRNAseq v2 reagents, (barcode sets C and D, PerkinElmer NOVA-5130-14 and NOVA-5130-15) and 16 cycles of PCR. Individual and pooled library quality was assessed on an Agilent Bioanalyzer and quantified with a Qubit spectrophotometer. Sequencing was performed by the UCL Cancer Institute, using an Illumina HiSeq 2500 instrument (paired-end 50bp) for the *Foxo-Aop*^*ACT*^*-Pnt*^*P1*^ epistasis experiment, and a NextSeq 500 (paired-end 75bp) for the *Pnt*^*RNAi*^ experiment.

### cDNA and qRT-PCR

cDNAs were made from the polyA RNA preps that were prepared for sequencing, using SuperScript II Reverse Transcriptase (Thermo 18064014) and OligoDT. qRT-PCR was performed on an Applied Biosystems QuantStudio 6 Flex real-time PCR instrument with Fast SYBR Green PCR Master Mix (Thermo Fisher), with primers supplied by EuroFins Genomics (all oligo sequences in S1 Supplementary Information), relative to a standard curve comprising a pool of all samples and the instrument's standard PCR cycle.

### Metabolic assays

TAG was measured as in [45] in whole adult *S_1_06; UAS-Pnt*^*P1*^ flies following one week of RU_486_ feeding. Briefly, flies were CO_2_-anaesthetised, weighed on a microbalance, and immediately flash-frozen in liquid N_2_. Flies were thawed in ice-cold TEt buffer (10 mM Tris, 1 mM EDTA, 0.1% v/v Triton-X-100) and homogenised by shaking with glass beads (Sigma G8772) for 30s in a ribolyser at 6500 Hz. Aliquots of homogenates were heated to 72°C for 15m to neutralise enzymatic activity, then spun 1m at 4500g and 4°C to pellet debris. Triglyceride was measured by treating 5 μl sample with 200 μl Glycerol Reagent (Sigma F6428) for 15m at 37°C and measuring absorbance at 540 nm, then incubating with 50 μl Triglyceride Reagent (Sigma F2449) for 15m at 37°C and re-measuring absorbance at 540 nm, calculating glycerol content in each reading, then quantifying triglyceride content as the difference between the first and second measurement.

### Data analysis

Sequence libraries were quality-checked by FastQC 0.11.3, duplicate reads were removed using Je 1.2, and reads were aligned to *D*. *melanogaster* genome 6.19 with HiSat2 2.1. Alignments were enumerated with featureCounts 1.6. All downstream analyses were performed in R 3.3.1. The gut and fat body were analysed in parallel. In the RNA-Seq experiment analysing *Foxo-Aop*^*ACT*^*-Pnt*^*P1*^ epistasis, genes with no counted transcripts were excluded (S1 Supplementary Information). In the subsequent *Pnt*^*RNAi*^ experiment, genes were filtered by the same criteria and any genes that were not analysed in the first experiment were also excluded. Read counts are given in S1 Supplementary Information. The transcriptomic effect of RU_486_ feeding was established for each individual genotype in the experiment, using DESeq2 at a false discovery rate (IHW) of 10%. To identify correlated effects amongst genotypes, sets of shared targets were formed as unions of DE gene sets from individual genotypes. Log2 fold-change values (Figs 1–4) were plotted from the DESeq2 output. Three-way epistatic interaction amongst TFs were identified by fitting models of the form

$$y^{i}∼genotype+RU_{486}+block+genotype:RU_{486}$$

where *block* represented experimental replicate. The tripartite interaction of *Foxo*, *Aop*^*ACT*^ and *Pnt*^*P1*^ was identified by applying the model to all genes across all experimental conditions, and isolating genes with a significant *genotype*:*RU*_*486*_ term.

GO analysis was performed using the TopGO package, applying Fisher’s test with the *weight01* algorithm. Principal Components Analysis was performed on read counts of these genes following a variance-stabilizing transformation. To characterise gene-expression correlates of principal components, loadings onto principal components were extracted using the dimdesc function from the FactoMineR library, and GO analysis performed as previously. Transcripts of genes annotated with enriched GO terms were then plotted per term by centring variance-stabilised reads to a mean of zero and plotting against PC values per sample. Heatmaps were plotted using the heatmap.2 function from the gplots library, ordering rows by hierarchical clustering by Ward’s method on Euclidian distance, and scaling to row.

Fly lifespan data were analysed using log-rank tests in Microsoft Excel or Cox Proportional Hazards in R for the interaction of sugar and *Pnt*^*P1*^ expression. Worm lifespan data were analysed by log-rank tests in JMP.

Luciferase reporter data were normalised by taking the ratio of firefly luciferase to renilla luciferase signal and, for each promoter, taking the median reporter signal in the absence of FOXO and AOP^ACT^ as the start value, then calculating fold-change (i.e. difference in start and end values, divided by start value) for each sample. To assess the interaction of FOXO and AOP with promoters’ complements of TF-binding motifs, these normalised data were analysed by fitting a linear model of the form

$$y∼FRE*EBM*FOXO*AOP^{ACT}$$

in which *y* was the natural log of fold-change+1, FRE and EBM represented the TF-binding complement, and FOXO and AOP^ACT^ represented co-transfection with *pAHW-Foxo* or *pAW-Aop*^*ACT*^. By the same approach, the interactive effect of PNT^P1^ and AOP^ACT^ were assessed by fitting a linear model of the form

$$y∼PNT^{P1}*AOP^{ACT}$$

in which y represented the natural log of fold-change+1.

The effect of *Pnt*^*P1*^ overexpression on TAG and lifespan responses to nutrient stress (starvation or high-sugar diet) were analysed by a model of the form

$$y∼RU_{486}*diet$$

where y represented TAG normalised to unit weight in a linear model, or survival in a Cox Proportional Hazards model (survival library).

## Supporting information

S1 Fig Venn diagram showing overlap between the unions of *Foxo* and *Aop*'s regulons in the gut and fat body. Numbers represent set and intersection size.
(EPS)

S2 Fig Replicate experiments of results shown in Fig 2A. Results were qualitatively consistent in each of the three replicates: AOP^ACT^ both moderates and synergises with transcriptional activation by FOXO on synthetic promoters containing combined ETS-binding motifs (EBMs) and FOXO-responsive elements (FREs), upstream of a basal *Adh-Firefly*^*luciferase*^ reporter. Activity is shown following normalisation to internal *Renilla*^*luciferase*^ controls, and calculation of fold-change over the median expression of each reporter in the absence of FOXO and AOP^ACT^. A significant four-way interaction of promoter and TF combination (i.e. FRE:EBM:FOXO:AOP) was detected in each experiment and in the pool. Full statistical analysis for each replicate experiment and their pool in S1 Supplementary Information.
(EPS)

S3 Fig Expression of transcripts subject to the 3-way *Foxo-Aop-Pnt* interaction (Fig 3D and 3E and S1 Supplementary Information), and annotated with significantly enriched GO terms (the five categories with lowest enrichment p-values), plotted over the first principal component of the expression matrix for each tissue (correlated to lifespan). Expression values were derived by applying *DESeq2*'s variance-stabilising transformation to read counts, taking medians per transcript, and mean-sweeping values. Principal component values are shown in Fig 3D and 3E.
(EPS)

S4 Fig *PntP1* overexpression in the gut and fat body does not accentuate loss of overall body mass upon starvation. Linear model: RU_486_, F_1,19_ = 4.9, p = 0.04; starvation F_1,19_ = 465.55, p = 8e-15; RU_486_:starvation F_1,19_ = 2.45, p = 0.13).
(EPS)

S5 Fig Supplementary lifespan analysis—limiting ETS TFs ubiquitously and with *S_1_06*. Corresponding percentage changes in lifespan and associated p-values are given in S1 Supplementary Information and Fig 6B. **(A-F)** Expressing RNAi against each ETS TFs other than *Aop* and *Pnt*. *Ets21C* or *Ets97D* knockdown extended lifespan. **(G-L)** Expressing RNAi against the same set of ETS with the ubiquitous *DaGS* driver. *Ets21C or Eip74EF* knockdown extended lifespan. **(M-N)** Heterozygous and homozygous mutants of *Ets21C* were long-lived. Note that results from one experiment, with the same control population, are plotted in the two separate panels. **(O-P)** Knocking down *Pnt* with the ubiquitous *DaGS* or *ActGS* drivers limits lifespan, noting that an equivalent result is observed in S6I Fig, indicating that strong knockdown in the wrong adult tissues is costly. **(Q-R)** Confirmation with *ActGS* that neither *Ets65A* nor *Ets98B* limit lifespan.
(EPS)

S6 Fig Expressing *Foxo* **(A-C)** or limiting ETS activation (*Aop*^*ACT*^ **(D-F),** *Pnt*^*RNAi*^ **(G-I),** *Ets21C*^*RNAi*^ **(J-L)**) has cell type-specific effects in the gut. TFs were chosen for experimentation based on strength of lifespan extension with *S106*, excluding *Ets97D*^*RNAi*^ due to its relatively mild effect therein.
(EPS)

S7 Fig Impacts of neuronal ETS/Foxo signalling on lifespan. **(A)** Neuronal *Eip74EF*^*RNAi*^ extends lifespan. **(B)** Neuronal *Pnt*^*RNAi*^ does not impact lifespan. **(C-D)** Neuronal *Aop*^*ACT*^ or *Foxo* are toxic.
(EPS)

S8 Fig Receptor tyrosine kinases (RTKs) known to function upstream of ETS TFs also have tissue-specific impacts on lifespan. **(A-B)** Expressing dominant-negative forms of the insulin receptor *InR* or epidermal growth factor receptor *EGFR* in gut and fat body with *S106*, extended lifespan. **(C-D)** Expressing the same constructs using *GS5966* reveals differentiation between impacts of insulin and EGF signalling in the gut and fat body. **(E-F)** Neither RTK construct extended lifespan when expressed using the ISC-specific driver *GS5961*. **(G-H)** Dominant-negative *InR*, but not *EGFR*, mildly extends lifespan when expressed under control of *TiGS*.
(EPS)

S1 Supplementary Information Containing supplementary tables, with explanatory key on the first sheet to all subsequent sheets. (XLSX)
